# Supplementary material for: Sustained-input switches for transcription factors and microRNAs are central building blocks of eukaryotic gene circuits
Source: Genome Biol. 2013 Aug 23;14(8):R85. doi: 10.1186/gb-2013-14-8-r85 (PMC4054853; doi:10.1186/gb-2013-14-8-r85)
Supplement: Additional file 5 — HTML Browsable Motif Output. Zipped folder containing all WaRSwap and FANMOD motif output, viewable in a web browser. [file gb-2013-14-8-r85-S5.ZIP › HTML_browsable_motif_output/WaRSwap_all_tair9/sigs_WSWRSLDraw-2000_all.pvals.heatmaps.html/motif_id_238_011101111_tftype_all_upstream_-2000_0.html]

```
BG_MODEL = Rnew2
MOTIF_ID = 238_011101111
TF_TYPE = all
UPSTREAM = -2000_0


PVals
FN_0.2	FN_0.4	FN_0.6	FN_0.8
dg_60.genes	0.108	0	0.026	0.001
dg_70.genes	0.061	0	0.035	0.001
dg_80.genes	0.406	0.025	0.009	1

ZScores
FN_0.2	FN_0.4	FN_0.6	FN_0.8
dg_60.genes	1.231	7.312	-0.162	-0.022
dg_70.genes	1.664	8.525	-0.181	-0.022
dg_80.genes	-0.345	1.673	-0.095	NA

StDevs
FN_0.2	FN_0.4	FN_0.6	FN_0.8
dg_60.genes	3.815	1.01	0.17	0.022
dg_70.genes	3.27	0.877	0.204	0.022
dg_80.genes	1.496	0.487	0.094	NA
```
